# Novel Gene Regulation in Normal and Abnormal Spermatogenesis

**DOI:** 10.3390/cells10030666

**Published:** 2021-03-17

**Authors:** Li Du, Wei Chen, Zixin Cheng, Si Wu, Jian He, Lu Han, Zuping He, Weibing Qin

**Affiliations:** 1 The Key Laboratory of Model Animals and Stem Cell Biology in Hunan Province, School of Medicine, Hunan Normal University, 371 Tongzipo Road, Changsha 410013, China; duli0809@126.com (L.D.); cweixy3@126.com (W.C.); chengzizi09@126.com (Z.C.); ws912731032@163.com (S.W.); hj604195132@126.com (J.H.); 2 The NHC Key Laboratory of Male Reproduction and Genetics, Family Planning Research Institute of Guangdong Province, Guangzhou 510600, China; sysuhanlu@126.com

**Keywords:** genes, regulation, spermatogenesis, functions and mechanisms, male infertility

## Abstract

Spermatogenesis is a complex and dynamic process which is precisely controlledby genetic and epigenetic factors. With the development of new technologies (e.g., single-cell RNA sequencing), increasingly more regulatory genes related to spermatogenesis have been identified. In this review, we address the roles and mechanisms of novel genes in regulating the normal and abnormal spermatogenesis. Specifically, we discussed the functions and signaling pathways of key new genes in mediating the proliferation, differentiation, and apoptosis of rodent and human spermatogonial stem cells (SSCs), as well as in controlling the meiosis of spermatocytes and other germ cells. Additionally, we summarized the gene regulation in the abnormal testicular microenvironment or the niche by Sertoli cells, peritubular myoid cells, and Leydig cells. Finally, we pointed out the future directions for investigating the molecular mechanisms underlying human spermatogenesis. This review could offer novel insights into genetic regulation in the normal and abnormal spermatogenesis, and it provides new molecular targets for gene therapy of male infertility.

## 1. Introduction

Spermatogenesis is an elaborately organized process inwhich diploid spermatogonial stem cells (SSCs) differentiate into spermatocytes and haploid spermatozoa. This process is collaborated by somatic cells in the testis, including Sertoli cells, peritubular myoid cells, and Leydig cells. In the past decades, we and others have revealed the molecular mechanisms underlying rodent spermatogenesis. In recent years, several new technologies, e.g., single-cell RNA sequencing and RNA deep sequencing, have been developed, making it feasible to identify more and more novel genes that are involved in the regulation of rodent and human spermatogenesis. In the current review, we addressed the functions and mechanisms of key novel genes in controlling the mitosis and meiosis of rodent and human male germ cells. We also discussed the roles of genes from the normal and abnormal niche of the testis and the perspectives in this field.

## 2. Novel Gene Regulation in the Fate Decisions of Human SSCs

Human SSCs self-renew to maintain the pool of stem cells in the testis and differentiate into mature spermatozoa. The fate determinations of SSCs, including the self-renewal, differentiation, and apoptosis, are essential for the maintenance of human spermatogenesis [1]. Notably, human and rodent SSCs have the great plasticity, as evidenced by the findings that they are able to become embryonic stem cell-like cells that differentiate into all cell types of three germ layersand can be transdifferentiated to other cell lineages [2,3,4,5,6]. As such, human SSCs could have significant applications in both reproductive and regenerative medicine. Human SSCs have the phenotypic characteristics of SSEA4^+^, CD49^+^, GPR125^+^, and c-Kit^neg/low^, which makes it feasible for human SSC enrichment, self-renewal, clonal expansion, and differentiation [7].

As a novel method, single-cell RNA sequencing has been employed to reveal the key genes and critical cell signaling pathways of human SSCs. The testicular cells were obtained from male aged from 2 days [8] to 60 years old [9] and separatedby MACS (c-KIT^+^cells [10], c-KIT^+^/SSEA4^+^cells [11], ITGA6^+^cells [8]), by FACS (GPR125^+^/DDX4^+^cells [9]), or StaPut [12], and they were analyzed by 10X Genomics [8,10,12,13] or/and Fluidigm C1 [11,12]. These studies have revealed the phenotypic characteristics of human spermatogonia from infancy to adulthood, indicating the heterogeneous features of these cells. Three neonatal human SSC clusters—the primordial germ cell (PGC)-like cells, PreSPG (prespermatogonia)-1, and PreSPG-2—are the most undifferentiated cells, and they do not proliferate or undergo slow proliferation [8]. Infant spermatogonia are most similar to adult SSCs State 0 (FGFR3^high^TSPAN33^high^SSEA4^low^), and they specifically express TBX3 and HOXA3 [10]. Puberty spermatogonia can be classified to the undifferentiated SPG (spermatogonia) and the differentiating SPG, which are marked byUTF1 (undifferentiated embryonic cell transcription factor 1) and KIT, respectively [13]. Meanwhile, distinct adult human SSC clusters (2–5) have been identified, which provides clear evidence for the heterogeneity of human SSCs [8,9,10,11]. TheKIT (differentiation marker) andMKI67 (proliferation marker) are specifically expressed during or after State 2, indicating State 0 and State 1 (UTF1^high^/GFRA1^low^ or GFRA1^high^/UTF1^low^) represent the quiescent SSC states, whereas State 2 indicates an initial differentiation and self-renewal state. *TCF3* is expressed in States 0 and 1, suggesting that it may contribute to retain the undifferentiated state of human SSCs [10]. Similarly, 2% of AdVac, a small subpopulation of A_dark_spermatogonia with nuclear rarefaction zone, seems to be entirely quiescent cells with high expression of UTF1 and lacking GFRA1 [14]. The undifferentiated spermatogonia remain dormant or slowly self-renew during infancy and pre-puberty, and they develop to the limited and incomplete SSC differentiation in early puberty and then establish a balance between the self-renewal and differentiation in the phases of adulthood.

**Figure 1:**
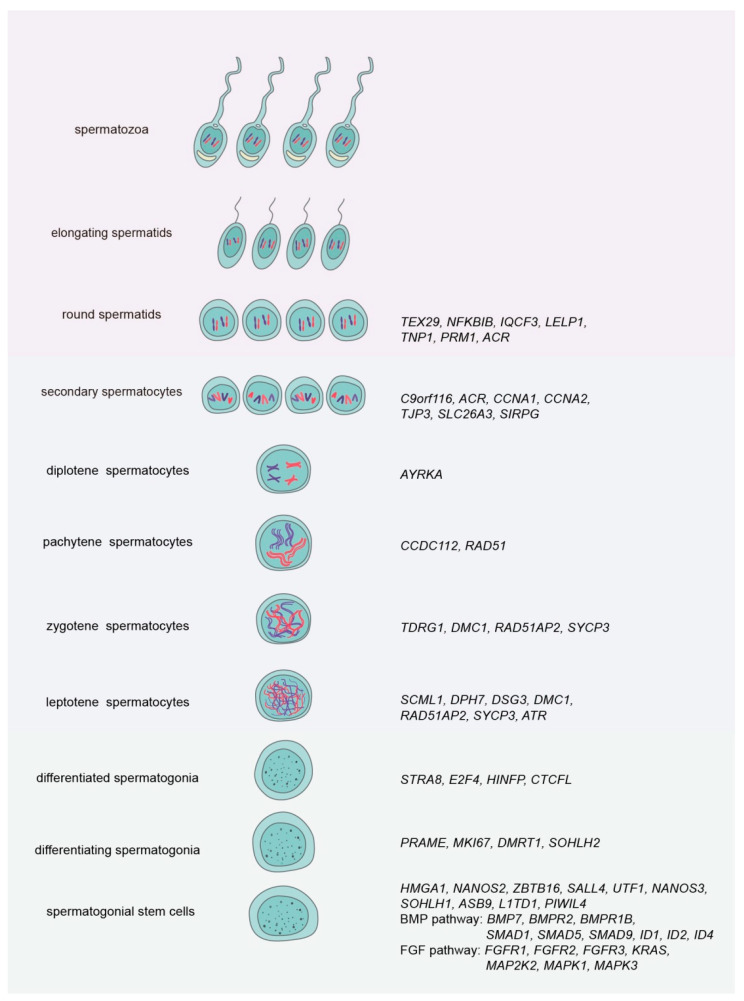
Expression of new genes in human spermatogenesis by single-cell RNA sequencing. Left and middle panels: the main types of human male germ cells; right panel: new genes and signaling pathways identified in each type of human male germ cells.

As shown in Figure 1, single-cell RNAsequencing also reveals numerous signaling pathways for human SSCs, including FGF pathways (e.g., *FGFR1* (fibroblast growth factor receptor 1), *FGFR2*, *FGFR3*, *KRAS*, *MAP2K2*, *MAPK1*, and *MAPK3*) and BMP pathways (e.g., *BMP7*, *BMPR2*, *BMPR1B*, *SMAD1*, *SMAD5*, *SMAD9*, *ID1*, *ID2*, and *ID4*) [9]. Specifically, the surface markers *FGFR3*, *DSG2* (desmoglein 2), *PLPPR3* [15,16], and other genes have been identified as the novel signatures. It has been reported that PLZF (promyelocyticleukaemia zinc finger) inhibits the differentiation of mouse SSCs via binding to the promoter regions of differentiation-associated genes (*Kit*, *Stra8*, *Sohlh2*, and *Dmrt1*) [17]. Nevertheless, the roles and mechanisms of these genes in the fate decisions of human SSCs remain to be explored.

Interestingly, DNA hypomethylation at embryonic developmental genes (*SOX2*, *KLF4*, *SALL4*, *TCF3*, *MBD3*, *STAT3*, and *KLF2*) supports their epigenetic “poising” in human SSCs for embryonic expression, while the levels of core pluripotency genes (*OCT4* and *NANOG*) are transcriptionally and epigenetically repressed. ATAC sequencing reveals top 12 motifs, including *CTCF*, *DMRT1/6*, *CTCFL*, *NFY*, *PGR* (progesterone receptor), *G R*(glucocorticoid receptor), *SOX9*, *FOXP1*, *SOX3*, *FOXA2*, *SMAD2*, and *AR* (androgen receptor), are enriched in human SSCs. Through further analysis, stem cell transcription and signaling factors promote the transfer of glucose into cells, causing mitochondrial activation and transforming human SSCs from static condition to the differentiated state [11]. Beyond the coding genes, transposable elements (TE) and lncRNA (e.g., LINC01030) contribute to the balance of human SSCs as well [10].

Recently, we have demonstrated that *FOXP3* variants cause male infertility and FOXP3stimulates the proliferation and inhibits the apoptosis of human SSCs [18]. We have also revealed that PAK1 regulates the proliferation, DNA synthesis, and apoptosis of human SSCs through the PDK1/KDR/ZNF367 and ERK1/2 and AKT signaling pathway [19]. Additionally, we have found that *JAZF1* silencing decreases cell proliferation and DNA synthesis as well as increase the apoptosis of human SSCs [20]. In contrast, we have reported that the silencing of microRNA targets, namely, *KLF2* (kruppel-like factor 2) [21], *CBL* [22], and *NFIX* [23], results in theincrease of proliferation and DNA synthesis as well as the reduction of apoptosis of human SSCs. Notably, we have shown the PAK1/PDK1/miRNA-31–5p network in mediating the self-renewal and apoptosis of human SSCs, which illustrates the genes/miRNAs (genetics and epigenetics) for the regulation of human SSCs [19,20]. Collectively, our studies highlight the important functions of genes in determining the fate decisions of human SSCs and male fertility, and offer novel endogenous targets for gene therapy for male infertility.

## 3. Novel Gene Regulation in Fate Determinations of Rodent SSCs

The single-cell RNA sequencinganalyzed spermatogenic cells of mice, and 7031 genes were found to be involved in spermatogenesis, which shows the expression profiles ofthe prototypical mouse SSCgene signatures (*Ddx4*, *Gfra1*, *Id4*, *Nanos2*, and *Plzf*). Notably, it has identified a panel of novel genes as we summarized in Table 1. As a subset of the type A_single_spermatogonia, ID4^+^cells are thehighest population in neonatal mice, which comprise 2% of the undifferentiated SSCs in adulthood [24]. In mice lacking *ID4* expression, normal spermatogenesis is impaired due to the gradual loss of the undifferentiated mouse SSCs in adulthood. In vitro, wildtype mouse SSCs survive, but their proliferation ability is abolished due to the reduction of ID4 expression. These results indicate that ID4 is a marker of male germline stem cells and it is critical for the regulation of cellself-renewal [25]. Another gene signature, *Nanos2* is expressed in the self-renewing mouse SSCs and it maintains the stem cell property [26]. By contrast, *NEDD4* (an E3 ubiquitin ligase) targets *NANOS2* in mouse SSCs, which leads to cell differentiation [27].

Similarly, Wnt6/β-catenin and p38 MAPK signaling pathways by genes determine the fate of mouse SSCs. The Wnt6/β-catenin pathway specifically promotes mouse SSC proliferation (37), while *Eif2s3y* regulates the self-renewal of mouse SSCs via Wnt6/β-catenin signaling pathway [31]. In addition, *SHISA6* inhibits mouse SSC differentiation through Wnt/β-catenin signaling [32]. P38 MAPK-specific inhibitors decrease the mouse SSC self-renewal ability [33], indicating that the p38 MAPK pathway contributes to the survival of mouse SSCs. *FGF9* promotes mouse SSC proliferation by p38 MAPK signaling [34], while we have found that VEGFC/VEGFR3 signaling regulates mouse SSCproliferation via the activation of AKT/MAPK and Cyclin D1 pathway and mediates the apoptosis by affecting Caspase 3/9 and Bcl-2 [35]. *Foxo1* is necessary for SSC homeostasis and spermatogenesis initiation, and the combined deficiency of *Foxo1*, *Foxo3*, and *Foxo4* results in the severe impairment of mouse SSC self-renewal and complete differentiation disorder [36]. *GLIS3* (GLI-similar 3) is expressed in mouse SSCs. In *Glis3* knockout mice, nuclear translocation of *FOXO1* is inhibited and mouse SSC number is significantly reduced, which causes the severely impairedspermatogenesis [37].

Moreover, a number of genes play critical roles in the differentiation and maintenanceof mouse SSCs. *Trim28* (tripartite motif-containing 28) promotes the differentiation of mouse SSCs [38], while specific deletion of *TRIM71* results in the reduced number of undifferentiated spermatogonia and hinders the transition to differentiated state [39]. *DAZL* deficiency compromises the expansionand differentiation of spermatogonial progenitor cells by mediating extensive translation programs [40]. In the absence of *Pramef12*, the number of mouse SSCs is decreased, and low expression of SSC maintenance-related genes and a defective ability of differentiation are observed [41]. The Ras-cyclin D2 pathway regulates the balance between tissue maintenance and tumorigenesis in the mouse SSCs [42]. PAX7*^+^* mouse SSCs self-renew and produce extended clones that differentiate into mature spermatids [43]. Due to losing *Rhox10*, the number of mouse SSCsis dramatically reduced by mediatingspermatogonial differentiation and migration to the mouse SSC niche [44]. The reduced *Pou3f1* expression induces male germ cell apoptosis and the impaired mouse SSC maintenance [45]. *FXRα* establishes and maintains an undifferentiated germ cell pool by regulating the expression of pluripotency factors (e.g., *Lin28*) [46]. We have found that *STAT3* is a target of miRNA-20 and miRNA-106a that regulate the self-renewal of mouse SSCs [47], while *DND1* retains the stemness of SSCs by recruiting CCR4-NOT complex [48]. Together, these studies shed a novel light on gene regulatory mechanisms controlling mouse SSC fate decisions.

## 4. Novel Gene Regulation in Human and Rodent Other Germ Cells

The single-cell RNA sequencingreveals that human germ cells can be classified to several types of cells based upon the biochemicalphenotypes, namely, differentiating-SPG (spermatogonia) (*PRAME*, *MKI67*, *DMRT1*, and *SOHLH2*), differentiated-SPG (*STRA8*, *E2F4*, *HINFP*, and *CTCFL*), leptotene spermatocytes (*SCML1*, *DPH7*, *DSG3*, *DMC1*, *RAD51AP2*, *SYCP3*, and *ATR*), zygotenespermatocytes (*TDRG1*, *DMC1*, *RAD51AP2*, and *SYCP3*), pachytenespermatocytes (*CCDC112* and *RAD51*), diplotenespermatocytes (*AYRKA*), SPC7 (spermatocytes 7) (*C9orf116*, *ACR*, *CCNA1*, *CCNA2*, *TJP3*, *SLC26A3*, and *SIRPG*), and spermatids (*TEX29*, *ACR*, *NFKBIB*, *TNP1*, *PRM1*, *IQCF3*, and *LELP1*), as we showed in Figure 1. Specifically, *FGFR3*, *DSG2*, E3 ubiquitin ligase c-CBL, CTAG1A/B (cancer/testis antigen NY-ESO-1), UTF1, and SNAP91 (synaptosomal-associated protein 91 kDa homolog)havebeen regarded as specific biomarkers of human spermatogonia [16]. Some of these genes have been examined for their functions and mechanisms. As examples, *PRAMEF12* (preferentially expressed antigen of melanoma family member 12) and *Dmrt1* (doublesex-related transcription factor) influence the spermiogenesis by regulating the survival of human germ cells. In male mice, *Pramef12* gene ablation prevents spermatogenesis and leads to sterility, which can be rescuedby transgenic expression of *Pramef12*, and *Pramef12* deficiency leads to overall decrease of spermatogenesis-related gene expression [41]. *DMRT1* acts on spermatogonia, restricts retinoic acid response, directly inhibits *Stra8* transcription, and activates the transcription of spermatogonial differentiation factor *Sohlh1*, thus preventing the meiosis and promoting the development of spermatogonia [49].

Our group has foundthat a total of 4276 genes are differentially expressed in human undifferentiated spermatogonia and spermatogonia. Among them, 2123 genes are upregulated in the undifferentiated spermatogonia, whereas 2153 genes are upregulated in spermatogonia. Interestingly, sevenof these genes belong to the HOX family, suggesting that *HOX* genes play an important role in mediating the differentiation of mouse germ cells. Gene aggregation and enrichment analysis were used to predict the transcription factor targets of differentially expressed genes during spermatogenesis. Among them, *NFATs*, *SP1*, and *TCF3* have been identified in human spermatogonia, spermatocytes, and spermatids, respectively, and these transcription factors are considered to be key regulators of human spermatogenesis d [50].

Single-cell RNAsequencing also uncovers the criticalregulators (*Uchl1*, *Tcea3*, *Crabp1*, *Prdm9*, *Dmrtb1*, *Tex101*, *Hspa5*, *Stra8*, *Sycp3*, *Hormad1*, *Hormad2*, *Sycp1*, *Tex15*, and *Ly6k*) in the regulation of mouse spermatogonia [12,28,30], as we discussedin Table 1. Yet the specific functions and mechanisms of most of these genes remain unknown. It has been reported that *Fbxo47*, *Pparg*, and *Ccnb3* are involved in mouse spermatogenesis, and male mice lacking *Fbxo47* are completely sterile, as spermatogenesis is arrested before meiotic recombination [30]. In addition, *Fbxo47* defective spermatocytes are unable to form complete synaptonemal complexes, and the destruction of *Fbxo47* destabilizes TRF2, resulting in unstable telomere attachment and slow traversing through the bouquet stage [51], implicatingthe *Fbxo47* regulatory role in the early stages of meiosis prophase I.

On the other hand, multifunctional roles have been identified for *DDX5* and the REGγ*-P53-PLZF* pathway inspermatogonia. RNA helicase *DDX5* is expressed in spermatogonia, which can splice the key genes necessary for spermatogenesis, while it regulatesthe expression of cell cycle genes in undifferentiated spermatogonia to ensure cell proliferation and survival [52]. Notably, the interaction of *DDX5* and *PLZF* has been shown to be required for germline maintenance [52]. Ablation of the proteasome activator *REGγ* leads to male sterility, with a decrease in the number of PLZF-positive spermatogonia [53]. Further studies show that *REGγ* deletion significantly increases the abundance of testicular P53 protein and directly inhibits the transcription of *PLZF*, suggesting that the REGγ-P53-PLZF pathway regulates the maintenance of mouse spermatogonia [53].

Furthermore, *L3mbtl2*, *ZMYM3*, *Bruce*, *PP6*, *PHB*, *SKP1*, *Claudin 3*, and *Sam68* have essential roles during metaphase to anaphase transition of mousespermatogenesis by regulating the fate of pachytene spermatocytes. L3MBTL2 is highly expressed in pachytene spermatocytes, and specific ablation of *L3mbtl2* leads to abnormal spermatozoa, gradual decrease of sperm counts, and premature testicular failure in mice. In the leptotene spermatocytes, *L3mbtl2* deficiency results in an increase of *H2AX* deposition, crossover and synaptic defects at the pachytene stage of meiosis I, and apoptosis and degradation of male germ cells in aging mice [54]. Knockdown of *Zmym3* results in spermatogenesis arrests at the meiosis prophase Iand the increased number of apoptotic germ cells [55]. Conditional deletion of the *Bruce* gene in the male germ line causes the impaired spermatogonial maintenance and chromosomal abnormalities during meiosis. DNA fragmentation, the damaged homologous synapses, nonhomologous association, and rearrangement occur in *Bruce*-deficient spermatocytes [56]. Spermatocytes with *PP6* defects are blocked at the pachytene stage with accompanying apoptosis, and DSB repair and cross formation are defective, indicating that *PP6* promotes the repair of meiosis double-chain fracture [57,58]. Spermatocytes with *SKP1* gene defects assume premature desynapsis [59], while *Claudin3* controls the process of early mouse spermatocyte meiosis [60]. The splicing regulator *Sam68* is highly expressed in meiotic cells, and *Sam68^−/−^* mice produce few spermatids with obvious motor deficit and inability to fertilize eggs [61,62].

In spermatids of *Tdrd6* (tudor domain-containing 6)-deficient mice, the chromatid bodies (CBs) are severely damaged, and the development of round sperm to elongated sperm, namely, spermiogenesis, is cancelled [63]. Together with *Tdrd6*, *Tdrd7* identifies the key biogenic processes of CBs [64], and the TDRD1/6/7/9 localization in CBs depends on Tdrd5 [65]. *Atg5* mutant mice have malformation of sperm head, discontinuous middle appendage structure, abnormal acrosomal formation, spermatozoa individualization loss, which results in about 70% infertility [66]. Germ cell-specific *Atg7* KO mice are sterile due to acrosomal biogenesis defects [67], suggesting that *Atg7* is necessary for prolongation of sperm development, sperm individualization, and normal fertility in male mice. In addition to Tdrds and Atgs families, *Spata 6* [68], *HIPK4* [69], *Cdy1* [70], and *TAp73* [71] are essential regulators for sperm head shaping and motility through the interaction with myosin subunits, F-actin, histone Kcr, and CDKN2B, respectively.

N-6-methyladenosine (m(6)A) is the most common internal modification in eukaryotic mRNA and may ensure the coordinated translation of the different stages of spermatogenesis. *Mettl3* or *Mettl14* with *Vasa*-Cre leads to the loss of m(6)A and depletion of SSCs. Deletion of m(6)A distorts the translation of transcripts required for SSC proliferation/differentiation. The removal of *Mettl3* in germ cells severely inhibits the differentiation of spermatogonia and hinders the initiation of meiosis [72]. Combined deletion of *Mettl3* and *Mettl14* with *Stra8GFP*-Cre in late germ cells disrupts sperm formation [73]. YTHDC2 is a m(6)A binding protein, and its knockout mice are sterile, asmale germ cells do not get past the zygotic stage and *Ythdc2* is upregulated in testis in the beginning of meiosis [74], suggesting that *Ythdc2* may be involved in mouse meiosis. As a m(6)A eraser, *ALKBH5* specifically removes m(6)A from target mRNAs and controls male sterility in mice [75]. As such, m(6)A modification is a key mechanism for controlling the mRNA fate of posttranscriptional meiosis and haploid cells.

## 5. Novel Gene Regulation in Testicular Microenvironment

**Figure 2:**
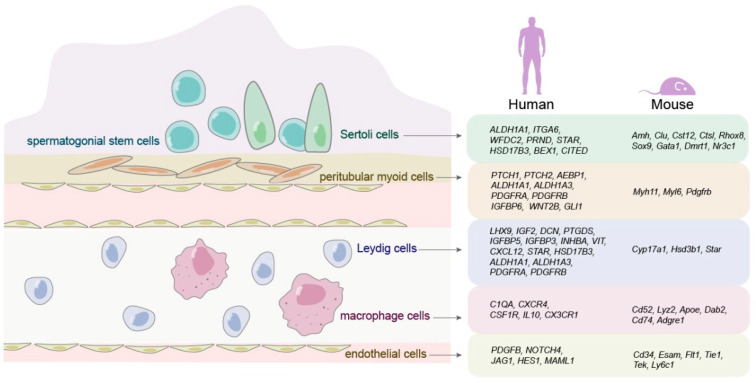
Expression of new genes in somatic cells of testis by single-cell RNA sequencing. Left panel: schematic illustration of major somatic cell types in the testis; right panel: new genes specifically expressed in specific types of somatic cells in human and rodent testis.

Spermatogenesis is precisely regulated by the microenvironment or the niche of the testis, which is mainly composed of the somatic cells as well as the growth factors and cytokines produced by the somatic cells. Single-cell transcriptome data of human and mouse testicular microenvironment uncover a set of new genes [8,28] as shown in Figure 2. In human, our group has found that *BMP6* accelerates the proliferation and represses the apoptosis of Sertoli cells via *DACH1* and *TFAP2A* activationand Smad2/3 pathway [76] and that *BMP4* promotes the proliferation of Sertoli cell through the Smad1/5 and ID2/3 pathway [77]. On the contrary, *GLI3* decreases the growth of human Sertoli cells [78]. In rodents, conditional ablation of *Mdm2* (murine double minute 2) in Sertoli cells results in a significant increase in apoptosis of these cells and male sterility [79]. Adult mice lacking *Insr* and *Igf1r* have the reduced testicular size and daily sperm production by 75% [80]. These studies illustrate that growth factors produced by human and rodent Sertoli cells are required for normal spermatogenesis and that their deficiencies cause male infertility.

The tight junctions between Sertoli cells in the blood–testis barrier (BTB) are essential for the migration and maturation of male germ cells during spermatogenesis. Conditional knockout of *Cx43* (connexin-43) mice assume the downregulated genes critical for mitosis and meiosis, e.g., *Stra8*, *Dazl*, and members of the *DM* (dsxand map-3) gene family, and the upregulated genes related to Sertoli cell maturation and proliferation [81,82]. The expression levels of cross-epithelial resistance and tight junctions are significantly increased in primary Sertoli cells of mice lacking *Cx43*. These results reflect the role of *Cx43* in regulating the function, composition, and dynamics of the BTB [83]. Inactivation of *Wt1* (Wilms tumor gene 1), specifically expressed in Sertoli cells, results in germ cell death and Sertoli cell-onlysyndrome (SCOS). The BTB is disrupted in *Wt1*-deficient testes. Meanwhile, polarity maintenance in Sertoli cells is controlled by *Wt1* via Wnt signaling pathway [84], and Wnt/β-Catenin signaling controls the spermatogenesis via Sertoli cell maturation [85]. The conditional deletion of *Uxt* [86], *Tspan8* [87], *Activin A* [88], *GATA4* [89], and *Cldn11* [90] in Sertoli cells results in the loss of male germ cells and incomplete structure of BTB, the smaller testis size, the reducedweight, and the eventually impaired spermatogenesis [87].

A network necessary for communication of niches is regulated by some new genes. Selective ablation of *AR* in mouse Sertoli cells completely blocks spermatogenesisat the meiosis stage [91]. Besides, GDNF (glial cell line-derived neurotrophic factor) produced by Sertoli cells and peritubular myoid(PM) cells is also critical for male germ cell development. Mice with specific knockout of *Gdnf* in PM cells results in sterility due to spermatogenesis disorder and the loss of undifferentiated spermatogonia [92]. Ablation of RNA-binding protein Ptbp2 in germcells leads to the disorder of filamentous actin cytoskeleton in Sertolicells [93]. The luteinizing hormone testosterone pathway regulates the self-renewal of mouse SSCs by inhibiting the expression of *WNT5A* in Sertolicells [94]. In addition, the translocation of genes over time is also necessary for spermatogenesis. RNA sequencing reveals that about 2939 genes in the Sertolicells assume dynamic stage-specific profiles, including cell cycle regulation, metabolism, and energy generation, RA synthesis, and biogenesis of the blood–testosterone barrier, which reflects the evolutionary role of Sertoli cells in controlling spermatogenesis [95]. Prior to puberty, MAST4 is localized to the Sertoli cells, and it is transferred to Leydig cells and spermatids throughout puberty [96]. Moreover, *Mast4* depletion leads to the increase of germ cellapoptosis, germ cell, and tubular structure loss and testis size reduction by the FGF2/ERM pathway [96].

## 6. Novel Gene Regulation in Abnormal Human Spermatogenesis

Around 10% of men suffer from infertilityworldwide, and the common causes of male infertility, e.g., idiopathic nonobstructiveazoospermia (NOA) andSCOS, may be derived from genetic defects [97,98]. Deletions or changes in the expression levels of genes have been shown to cause NOA, as shown in Table 2. There is a close association between NOA risk in Chinese Han males and common variations of *PRMT6*, *PEX10*, and *SOX5* [99]. Bi-allelic recessive loss-of-function variants in *FANCM* [100] or missense mutation of *WT1* [84] result in the NOA. In addition, NOA patients have a disorder of genomic methylation modification in testicular cells, with significant difference in the expression of reproduction-related genes [101]. Transcripts of *RARA*, *RXRB*, and *RXRG* are significantly reduced in patients with SCOS and maturation arrest (MA), but not in patients with spermatogenesis hypogenesis, suggesting that decreased levels of these genes are closely associated with the failure of SCOS and spermatogenesis MA [102]. New mutation in *USP26* is related toSCOS patients [103]. Our team has found differential expression of *LRP6* and *Cyclin D1* in Sertoli cells between SCOS and OA patients with normal spermatogenesis [104].

Notably, we have revealed that 10 of 300 NOA (3.3%) patients have *FOXP3* variants [18], which is 10 time higher than other gene variants in NOA patients, suggesting that *FOXP3* mutation is closely associated with male infertility. With regards to cryptorchidism, the *NR5A1* mutation appears to cause more severe forms of male infertility [126,127]. Exome sequencing of infertile men reveals three heterozygous *SYCP2* transcoding variants in cryptospermia and azoospermia [128]. These results may contribute to the development of new molecular indicators for spermatogenesis dysfunction and provide novel therapeutic targets for male infertility [102].

Meiotic arrest and abnormal morphology and movement of spermcaused by genetic abnormality are important factors, leading to spermatogenesis failure. Total exome sequencing shows that *M1AP* biallele mutation is a common cause of male infertility due to the cessation of meiosis and severe spermatogenesis damage [129]. In mutant homozygous patients, male germ cells are deficient in *SCAPER* expression, with early spermatogenesis defects and azoospermia, which leads to the complete loss of meiotic cells [130]. Expression of *1700102P08Rik* is downregulated in men with spermatocyte arrest [131]. Multiple morphological abnormalities of the sperm flagella (MMAF) is a severe form of asthenoteratozoospermia. Variants of several genes, including CFAP family members (*CFAP43*, *CFAP44* [132,133], *CFAP58* [134], *CFAP69* [135], and *CFAP251* [136]), *DNAH8* [137], *ARMC2* [138], *TTC21A* [139], and *QRICH2* [140], lead to multiple morphological abnormalities of the sperm flagella and primary male infertility. Mutation in *PMFBP1* [141] is involved in asthenospermia syndrome. Biallelic *SUN5* mutations [142] contribute to a severe teratozoospermia. The mutation of *DNAH17*, which enforces the heavy chain of sperm-specific outer dyneinarms and leads to flagellum instability and asthenospermia [143,144]. Besides the morphology, motility abnormalities of sperm caused by mutations in *DNAJB13* result in male infertility [145]. Meta-analysis of 6570 mutations indicates that germline methylation affects mutation rates. The mutation rate of each cell division is higher during early embryogenesis and primordial germ cell differentiation, while it is decreased significantly during post-pubertal spermatogenesis [146]. It is worth investigating whether the mutation in the above causes of abnormal spermatogenesis in men ismediatedby the methylation of the genes.

## 7. Perspectives and Future Directions

With the development of technologies, especially single-cell RNA sequencing, a number of novel genes critical for regulating spermatogenesishave been identified. Sperm-seq, a new way of simultaneously analyzing the genomes of thousands of individual sperm [147], may also offer new insights into gene regulation in spermatogenesis. Numerous genes are specifically expressed in male germ cells or somatic cells, but their specific functions and mechanisms remain to be explored further. The changes in the expression of these genes in time and location and the locus of gene expression are also important ways to investigate the roles and signaling pathways of spermatogenesis-related genes. After identifying the key genes, it remains to be determinedhow these genes are regulated by DNA or RNA methylation and other epigenetic regulators (e.g., miRNAs). Abnormal gene variants or mutation in male germ cells between different species, especiallyhuman, also warrant further exploration. These studies would provide new genetic regulators for human spermatogenesis and could offer novel targets for gene therapy of male infertility.

## Figures and Tables

**Table 1:** Novel genes involved in mouse germ cells by single-cell RNA sequencing.

Cells	Markers	Stages	Novel Genes	References
Germ cells	Ddx4	Undifferentiated spermatogonia	*Nanos2*, *Nanos3*, *Eomes*, *Pax7*, *Rhox10*, *Tspan8*, *Sall4*, *Sdc4*, *Bcl6*, *Taf4b*, *Lhx1*, *Dusp6*, *Epha2*, *Ptpn13*, *Pvr*, *Tcl1*	[12,28]
		Differentiatingspermatogonia	*Uchl1*, *Tcea3*, *Crabp1*, *Dmrtb1*, *Tex101*, *Hspa5*, *Stra8*, *Sycp3*, *Prdm9*, *Hormad1*, *Hormad2*, *Sycp1*, *Tex15*	[12,28]
		Early spermatocytes	*Meioc*, *Prdm3*, *Top2a*, *Smc3*	[29]
		Spermatocytes	*Piwil1*, *Pttg1*, *Insl6*, *Spag6*, *Tbpl1*, *Sycp1*, *Sycp2*, *Sycp3*, *Hzafx*	[12,28,29]
		Round spermatids	*Acrv1*, *Tssk1*, *Spaca1*, *Tsga8*, *Pgk2*, *Cd37*, *Cd63*, *Cd96*, *Cd177*, *Ranbp9*, *Morf4l1*, *Catsper3*, *Cstsper4*, *Spata25*, *Izumo1*	[28,29,30]
		Elongated spermatids	*Prm1*, *Prm2*, *Prm3*, *Tnp1*, *Tnp2*, *Hspa1l*, *Izumo3*, *Tssk6*, *Dnajb3*	[12,28]

**Table 2:** Novel genes involved in abnormal human spermatogenesis.

	Types	Novel Genes	References
	Maturation arrest	*CDY2*, *HSFY*	[105]
NOA	SCOS	*Pramef12*, *H3t*, *PLK4*, *CARF*, *FGF9*, *IGF1*, *ETV5*, *HnRNPL*, *PRPS2*	[41,106,107,108,109,110,111,112,113]
	Unclassified NOA	*TDRD7*, *TDRD9*, *TEX15*, *DMC1*, *DGCR8*, *FANCM*, *DDR1*, *SAM68*, *RanBP3*, *RNF212*, *STAG3*, *NPAS2*	[100,114,115,116,117,118,119,120,121,122,123,124,125]

## Data Availability

Not applicable.
